# The discourse of generational segmentation and the implications for postgraduate medical education

**DOI:** 10.1007/s40037-013-0057-0

**Published:** 2013-04-16

**Authors:** Jamiu O. Busari

**Affiliations:** 1 Medical Residency Program, Department of Pediatrics, Atrium Medical Center, Henri Dunantstraat 5, 6401 CX Heerlen, the Netherlands; 2 Department of Educational Development and Research, Faculty of Health, Medicine and Life Sciences, University of Maastricht, P. O. Box 616, 6200 MD Maastricht, the Netherlands

**Keywords:** Generation, Postgraduate medical education, Physicians, Training, Health care

## Abstract

The growing demands for easily accessible, cost effective and efficient health care services are hindering many medical training programs in delivering well prepared physicians, equipped with the competencies to tackle new and complex health care problems. In addition to this, many medical institutions are finding it difficult to design curricula that would prepare today’s physicians adequately for the ongoing changes in health care. Targeted customer service is a growing phenomenon in health care, where healthcare institutions are operating as retail service providers, design experiences and deliver care around the convenience of consumers rather than the preferences of providers. Gradually finding its way into medical education, this concept entails investing in understanding the beliefs and values of consumers as a result of their different expectations and differences. Defined by the experiences that create common values among the members of a specific group, the discourse of generation segmentation has proven to be a helpful way of understanding consumer differences. There are four known generations currently impacting the pattern and distribution of healthcare services and in the coming decade, the future of medical education In this paper, medical education is re-examined in the light of this phenomenon of generation segmentation and whether today’s physicians are being effectively prepared to practice in a fast changing world. The analysis provided in this paper presents a recommendation for the medical curriculum of a new millennium based on the changing needs and expectations of different generations of consumers.

## Introduction

Health care delivery and health professions education are at major crossroads in many countries today. It is thought that due to increased demands for easily accessible and cost-effective health care services, today’s practising physicians and those in training may no longer be capable of delivering the sort of care 21st century patients (would) need [1]. Also, with the current pace and increasing complexity of the changes in health care, it is becoming increasingly difficult for care providers to effectively manage the health care needs of their communities [1]. Each year, it is estimated that about one million physicians are trained in more than 2,000 medical schools and an estimated $100 billion/year is spent on health professions education globally. In addition, the average cost of tuition for each graduate medical student is set at approximately $113,000/year with unit costs being highest in North America and lowest in China [2].

In North America and Europe, the average time spent in medical training to become specialist physicians is between 5 and 7 years, in addition to the 3–5 years spent in undergraduate medical school. The past few years have, however, witnessed a rethinking of current training practices and many think that the duration of the medical training is too long. They argue that medical training programmes can be shortened considerably without any serious consequences to the outcome of the programmes. It is thought that by fostering attention on the essential content of the programmes and forcing educational institutions to eliminate unnecessary and repetitious material in the curriculum, an average of 14 years of training (running through college to the speciality fellowship period) could significantly be reduced by approximately 4 years. Bearing in mind that the average medical student in the United States graduates with an estimated $160,000 in debt, 4 years less of medical school would be significant in lowering tuition debts of trainees [3].

## The discourse of generational segmentation

While the pressures on health care systems and the costs of care are rising, new challenges to the traditional system of health care delivery are also emerging. New stakeholders are entering the business of health care in many countries, disrupting and redefining how health care can be accessed [4]. Health care has also turned into a consumer market where patients, i.e. the consumers, have found ways to navigate the complexities of various health care systems by comparing service, quality, and costs of care [5]. As a result, health care institutions are operating as retail service providers, designing experiences and delivering care around the convenience of consumers rather than the preferences of providers. They have also had to invest in understanding the beliefs and values of the consumers they serve because of their different expectations from the health care system, i.e. targeted consumer services [6]. This phenomenon of ‘targeted customer service’ in health care is a growing concept within medical education and it is expected to influence and shape the future training of physicians considerably.

Gaining increasing popularity around the mid-1990s, the concept of generation segmentation has proven to be a helpful means of understanding consumer differences and how knowledge of this can be used to provide specific services. Although defining ‘generations’ is not an exact science (as the breakdown is subjective and generalized), marketers and journalists use these groupings in targeting their marketing to particular age groups. There is the tendency for disagreement about the time frames covered by the generations, the names given to them and probably over-generalizations of the personalities. Nonetheless, generations can be defined by the experiences that create common values among the members of a specific group. There are four generations that have been identified as currently impacting the pattern and distribution of a number of services that includes the health care system, namely the Greatest Generation, the Baby Boomers, the Gen Xers, and the Millennials [7–9].

## The four generations

The members of the *Greatest Generation*—1925–1944—went through the Great Depression and served during World War II. Characterized by duty and sacrifice, the Greatest Generation value credentials as an indication of expertise. They generally accept authority and tend to follow society’s rules. The *Baby Boomers*—1945–1964—on their part grew up amidst great prosperity and believe that they can change the world. They experienced the Civil Rights movement, the Vietnam War, and the sexual revolution. Unlike the Greatest Generation, Boomers have a history of challenging traditional institutions and values. They feel that institutions have failed to handle societal needs responsibly and believe that rules are to be followed only if they can deliver the goods, if not ‘they are made to be broken, or modified’. The *Gen Xers*—1965–1984—grew up in a difficult time socially and financially. While their Boomer parents were striving for self-fulfilment and monetary success, they were often ‘latch key’ children. Gen Xers trust themselves and their peers rather than corporations and are described as the most self-sufficient and sceptical generation. They are considered the most loyal employees and flatly reject the Boomers workaholic approach. They invest loyalty in a person rather than an establishment. Finally, the *Millenials/Gen Yers*—1985–2005—are considered to be the most threatened and protected generation in history. Compared with previous generations, Millennials are excessively insulated with an unprecedented amount of parental supervision and advocacy. They are collaborative, tolerant, and comfortable with speed and change, they feel at home with multi-tasking and multiple forms of electronic/digital media. Their perception of life and world events has been formed through the lens of digital technology, e.g. personal computers and Smartphones [10].

(Table 1) provides an overview of how technology, health care and consumer markets have been influenced by the phenomenon of generational segmentation.

**Table 1:** The different generations approach to technology, market selection and health care

Generation	Technology	Marketing	Health care
Greatest Generation 1925–1944	Grew up with radio and party lines, do not require many of today’s technological advancements Marvel at their grandchildren’s technological expertise; do not share the integral need for technology in their everyday life – *Is this necessary?*	Loyal consumers Will shop around if necessary Value quality over efficiency Standard options satisfy them; customization not always necessary/valued Trust credentialed institutions to provide them with what they need – *Give Me What I Need*	Believe in the doctor as a gatekeeper who will assist in navigating the health care system View the doctor as a trusted authority figure to make appropriate health care decisions for them High users of health care due to co-morbidities and end of life needs The Boomers (i.e. their children) increasingly making their health care decisions – *Direct Me*
Baby Boomers 1945–1964	The beta generation for much of today’s technology; also sceptical about technology as bringing many problems as solutions Expect that technology will be available to support the personalization they expect from all of their service experiences – *Beta Testers*	Customization of products/services is important Less concerned with reputations and credentials Value products and services that offer prestige i.e. ‘membership has its privileges’ Respond to messages/services that allow them to gain control of their time through personalization – *Pursue Me*	Active consumers of health care Interested in a physician’s credentials as well as a hospital’s reputation Likely to do extensive research, prior to visiting a doctor including internet searches Expect to engage in a dialogue about the care they or their parents receive; tend to focus on personal wellness and fighting the ageing process – *Engage Me*
Gen Xers 1965–1984	The first PC generation Technology is a necessity, helps them control their schedules and their children’s schedules. It is not uncommon to see two Gen X parents in a coffee shop beaming play dates to each other from their iphones or Blackberries – *Balancing Act*	Rely on peer referrals more than any other generations Look for personalization, options, and control of the sale – *What do My Friends Say?*	Expect health care to be designed around them and their family’s needs rather than an ordinary physician/patient relationship Distrustful of institutions and disdainful of hierarchy and authority In search of a free flow of information; want electronic health care records and will use them to become advocates for their own care Engage in online disease management programmes and virtual coaching sessions on a variety of health topics Prefer to be self sufficient in health care as in other areas of their lives Would rather learn to diagnose their child’s ear infection than wait an hour for a doctor to perform the same simple examination – *Educate Me*
Millennials 1985–2005	Techno savvy generation, digital natives Believe that electronic/digital connections with others are acceptable as face-to-face communication Use technology to build and expand social networks and constantly communicate with ‘friends’ With the advent of Facebook, the Internet, Twitter, YouTube, etc., the concept of friendship has evolved from the boy next door, to the girl across the globe – *The world is Technology*	Raised to be consumers; have very high brand awareness Expect high quality and easy to handle experiences Expect the buying experience to be interactive; want products that are customized and unique, sold by companies with an altruistic attitude Raised with a significant environmental consciousness, prefer socially responsible companies as their suppliers – *Interact with Me*	Primarily interact with the health care system through paediatrics, obstetrics & gynaecology and sports medicine They are looking for immediate interactions that are technology based They will not wait for appointments, surgery dates, etc. preferring real time, and individualized, problem resolution – *Connect With Me*

## Generational segmentation and medical education

Regardless of generation, consumers expect quality, continuity and easy access to good health care. Decisions about hospital and care providers, however, are influenced by their generational experiences. According to the Thomson-Reuters Pulse Survey, Boomers often selected physicians based on hospital affiliation. Gen Xers and Millennials on the other hand rely on recommendations from friends and family. The Greatest Generation and Boomers were the least likely to switch physicians while Millennials and Gen Xers, were likely to switch their primary care physician due to service experiential factors such as lack of confidence, relationship satisfaction, location of service facility and waiting times [7]. A Deloitte health care customer survey in 2009 showed that 16 % of their respondents had switched physicians in the last year, with 2 out of 3 switching because of dissatisfaction with the service they received’[5]. For Gen Xers and Millennials service is a critical retention tool for physicians.

So what do the observed trends in generation segmentation mean for the way physicians of the future should (and need to) be trained? Similar to the different behaviours towards health care, consumer markets and the use of technology, the different generations demonstrate peculiar attitudes towards education. While members of the Greatest Generation revere the institution of education as the source of all knowledge, conform to rules and regulations and tend to experience having failed if and when feedback is offered, members of the Millennial generation, and to a lesser degree the Gen Xers, thrive on immediate and continuous feedback, feel insecure without it and expect to be acknowledged based on how big their social network followers are. Furthermore, members of this group increasingly turn to channels outside the traditional educational establishment for the source of their information and knowledge. Baby Boomers on their part question the legitimacy of authority feeling that the source of reliable knowledge or information begins and ends with them. They would happily stick to jotting notes on paper rather than use a ‘savvy and fault prone’ ipad tablet and they are the ones who tend to populate the category of ‘late adopters’ when it comes to utilizing modern technology (Table 2).

**Table 2:** The different generations relationship with (medical) education

Educational characteristic	Greatest Generation 1925–1944	Baby Boomers 1945–1964	Gen X’ers 1965–1984	Millennials 1985–2005
Authority	Conformers, authority rules Command and control	Usually uncomfortable with authority figures Question legitimacy of authority	Comfortable with authority Not intimidated by authority	Believe respect must be earned Question authority
Signs of respect	Revere authority/superiors Give special treatment	Revere authority/superiors Give special treatment	Demand authority Expect to be held in esteem Expect to be listened to based on earned professional/academic achievement	Demand authority Expect to be held in esteem Expect to be listened to based on the number of contacts/followers in network e.g. Twitter, Facebook
Source of knowledge	Personal experiences Storytelling Trial and error	Books and libraries Microfilms Experts/intellectuals Experiments	Electronic media, E-books CD ROMs Online information/databases	WWW, Wikipedia Google, You Tube Social networks
Favoured learning approach	Apprenticeship	Lectures Pen and paper Slides/overhead sheets	Emails PowerPoint presentations	Simulations (Serious) gaming You Tube instructions Virtual teaching on the web
Reaction to feedback	May feel insulted by continuous feedback	May feel insulted by continuous feedback	Feel at home with feedback Not dependent on immediate and continuous feedback	Thrive on immediate and continuous feedback Feel insecure without feedback
Key value	Feeling valued	Feeling valued	Feeling valued	Feeling valued

The discourse of generational gaps and its impact on the training of physicians also provides a different perspective for the ongoing transitions in (postgraduate) medical education. Similar to initiatives in health care and marketing, awareness of the phenomenon of generational segmentation and how it defines the behaviour of members within the different generations can and should be used in understanding the educational needs of the ‘Millennial’ physician in training [10]. It should be applied in defining how future medical curricula need to be designed so that they match the growing and changing health care needs in society. Especially when the expectation is that by the year 2030, an increase in the number of Gen Xers and Millennial patients, and to a lesser degree Baby Boomers, would cause a change in the demand and utilization of health care services. These changes are already forcing health care professionals to change the way they work.

## Conclusion

In conclusion, the changing trends in health care continue to pose new challenges for the way health care is currently organized and the way physicians would need to be trained. As physicians increasingly have to work with other professionals in health care teams or integrated care systems, the education of physicians would have to be modified to match the desires and values in a new community of health consumers [11, 12]. Based on these insights on generational differences, there is going to be a need for a new sort of physician (the ‘Millennial physician’), with a different set of skills and competencies than the current medical training programmes offer (Box 1). The duration of medical training would also need to be shorter than it currently is, due to increased costs involved with the training and from the redundancies currently being witnessed in many training programmes. The medical curriculum that would be needed would be one that focuses on developing more general, collaborative and community-specific competencies in physicians and made up of linear and modular rotations that are combined in remote settings, far from the mother institution. Finally, the end product of the training programmes, i.e. the Millennial physician, would be the digital native practitioner who can easily navigate his way through the vast array of web-based, electronic and mobile technology using these not only as a major means of communication, but also as the primary access to (medical) information and resources on the web.

**Box 1:** Characteristics of the Millennial physician

Expert in the science of medicine and the (interrelationships between) social sciences and humanities related to clinical care	
Skilled in communications, care giving and interpersonal relationships including patient advocacy and cultural sensitivity	
Is a professional, including being ethical and functioning as a member or leader of a team	
Life-long learner, able to reflect and evaluate self, and improving based on practice, experience and feedback	
Knowledgeable about the health care system, including the principles of economics, public health, management, quality assurance and patient safety	
